# Social determinants of health inequalities: towards a theoretical perspective using systems science

**DOI:** 10.1186/s12939-015-0205-8

**Published:** 2015-08-25

**Authors:** Saroj Jayasinghe

**Affiliations:** Department of Clinical Medicine, Faculty of Medicine, University of Colombo, Kynsey Road, Colombo, 8 Sri Lanka

## Abstract

A systems approach offers a novel conceptualization to natural and social systems. In recent years, this has led to perceiving population health outcomes as an emergent property of a dynamic and open, complex adaptive system. The current paper explores these themes further and applies the principles of systems approach and complexity science (i.e. systems science) to conceptualize social determinants of health inequalities. The conceptualization can be done in two steps: viewing health inequalities from a systems approach and extending it to include complexity science. Systems approach views health inequalities as patterns within the larger rubric of other facets of the human condition, such as educational outcomes and economic development. This anlysis requires more sophisticated models such as systems dynamic models. An extension of the approach is to view systems as complex adaptive systems, i.e. systems that are 'open' and adapt to the environment. They consist of dynamic adapting subsystems that exhibit non-linear interactions, while being 'open' to a similarly dynamic environment of interconnected systems. They exhibit emergent properties that cannot be estimated with precision by using the known interactions among its components (such as economic development, political freedom, health system, culture etc.). Different combinations of the same bundle of factors or determinants give rise to similar patterns or outcomes (i.e. property of convergence), and minor variations in the initial condition could give rise to widely divergent outcomes. Novel approaches using computer simulation models (e.g. agent-based models) would shed light on possible mechanisms as to how factors or determinants interact and lead to emergent patterns of health inequalities of populations.

## Introduction

Health outcomes are increasingly perceived from a systems approach that is more holistic and non-reductionist [1]. The author in a recent paper extended the systems approach to incorporate principles of complexity science and to conceptualize population health outcomes as an emergent property of a dynamic and open, complex adaptive system [2]. The current paper explores these themes further and applies the principles of systems approach and complexity science (i.e. systems science) to conceptualize social determinants of health inequalities (SDHI). The paper begins with a brief overview of the existing models of SDHI, and then proposes a two step approach to remodel our perspectives and outlines possible implications.

### Current conceptualization of SDHI

Interest in studying social influences of population health outcomes dates back to at least the 19th century. Pioneering researchers in this area include Rudolf Virchow who reported on the role of political economy and poverty in causing an epidemic of plague in Upper Silesia of Prussia, and Friedrich Engels on the link between high mortality and poor living conditions of the working class in England [3]. Subsequently Salvador Allende’s work in Chile attempted to show the role of social and political factors in generating health inequalities in populations [3].

More recent expansion of a population-based approach to inequalities includes the work of Geoffrey Rose in the 1980’s [4]. He distinguished between causes of incidence of a population group and causes of disease in individuals. The extreme example he proposes was to assume that every member of society smoked 20 cigarettes a day, which if investigates through cohort studies and case-control studies, will lead to the conclusion that incidence of lung cancer is determined by genetic predispositions. These smoking patterns are a reflection of social norms, values, traditions and their customs, in a historical context. Thus, societies with lower per-capita rates of smoking have a significantly lower incidence of lung cancer. The balances or imbalances of norms and social structures within the population lead to distinct patterns of individual behaviours in smoking rates or varying rates of disease prevalence or incidence among social groups, i.e. inequalities. Sick individuals represent the extremes of the population mean.

Recent literature uses the term social determinants of health inequalities (SDHI) to denote contexts, social norms, social structures, and their determinants. Three main pathways have been advanced to explain the generation of health inequalities from the social environment [5].

- “Social selection”, or social mobility which implies that health determines socioeconomic position, rather than the reverse. Thus, healthier persons will move towards better socioeconomic positions, compared to less healthier, leading to inequalities.
- “Social causation” proposes that a range of unequally spread material, psycho-social and behavioural factors, give rise to inequalities in health outcomes [6, 7]. Material factors include varying income levels and investments across infrastructure beneficial to the community. Psychosocial factors are the chronic stresses that arise from perceptions and experiences of personal status in an unequal society. Behavioral factors are, for example, the higher rates of smoking observed in poorer groups that lead to differential rates of diseases and mortality [8, 9].
- A "life course perspective" suggests that a multitude of factors across the life span (e.g. maternal malnutrition during fetal period, poor educational facilities in childhood, occupations with physical hazards etc.) determine and manifest disease trends observed over time. The ecosocial approach attempts to integrate these ecological, social and biological factors in disease causation through a dynamic process of their ‘embodiment’ i.e. “we literally incorporate biological influences from the material and social world” [10, 11]. There are several biological pathways that explain how social environment gets ‘embodied’ on health. One is the ‘stress biology approach’ whereby stress leads to chronic stimulation of the hypothalamo-pituitary-adrenal axis and the resultant hormonal changes increase risks of hypertension, insulin resistance, and hyperglycaemia [6]. The other relates to the advancing field of epigenetics, i.e. non-genetic modifiers of gene expression that are transmitted to future generation. They provide an explanation as to how environmental effects convert to lasting physiological changes and behavioral changes. An example is how psychological stress alters DNA methylation and histones in stress-sensitive brain regions such as the hippocampus, amygdala, and prefrontal cortex. These changes modulate subsequent gene expression [12]. Another pathway is through modifications of telomeres in dividing somatic cells. Telomeres are DNA repeat sequences, which together with accompanying telomere-binding proteins, cap and protect chromosome ends. Their length shortens with each cycle of replication, which is an expression of accelerated ageing as this process ultimately leads to a loss of capacity to replicate. It has been shown that lower socio-economic groups demonstrate shortening of the length of telomeres, thus suggesting that these groups acquire features of accelerated ageing with its consequences [13, 14].

The Commission of SDH (CSDH) re-conceptualizes health inequalities by integrating these theories and concepts further [5, 15]. They describe SDHI to have a context, structural mechanisms and socioeconomic positions of individuals. It assumes a crucial role for the “context” which includes social systems (e.g. education system, labour market), culture (e.g. racism and caste) and political systems (e.g. structure of the state, redistributive policies). The structure of the state in relation to welfare and re-distribution of wealth is recognized as a dominant institution. The ‘context’ should be viewed as a dynamic concept, having a historical past, a present and future trajectory. Structural mechanisms that are rooted in institutions and processes within the context generate stratifications in society according to socio-economic position, income or wealth, educational achievements and access, occupation, gender, race/ethnicity and other dimensions. These are inter-related dimensions and could act as proxies for each other. For example, in a heavily market-driven individualized society, incomes or wealth are good proxy indicators for socio-economic position. The socioeconomic position in turn is a key stratifier in most contemporary societies and reflects a hierachical system consisting of power, prestige and access to resources.

The SDHI operate through a group of intermediary determinants to mould health outcomes. The main groups of intermediary determinants of health are: material circumstances (e.g. quality of housing, exposure to pollution, financial means to purchase quality food, and work environment); psychosocial circumstances (e.g. levels of stress and social support); behavioural factors (e.g. rates of tobacco and alcohol consumption, nutrition and physical activity) biological factors (e.g. genetic predisposition to diseases in different population groups) factors) and the health system (e.g. access to quality care in populations). Increasingly, research evidence reports a widening range of influencing material circumstance (such as availability of safe water and sanitation, agricultural policies and food security, access to health and social care services, unemployment, under-employment and working conditions, access to housing, the living environment, access to education, and availability of transport) [15, 16]. Those holding higher positions in the hierarchies of social stratification (e.g. higher socio-economic position or most affluent) would hold an advantageous position in accessing resources, information and environments that are more favourable to better health outcomes.

## Limitations of current concepts of inequalities

An implicit and explicit recognition of an inter-related web of factors functioning as a system runs through the above discourse. Rose’s concept of causes of incidence in a population group, implies that the population functions as a cohesive ‘whole’ or system, rather than being a mere collection of independent individuals. Similarly, the concept of SDHI proposed by the CSDH describes a system that consists of elements such as, a context, structural mechanisms, and intermediary determinants. These are related both as influencers as well as through feedback mechanisms.

However, as with most concepts related to health outcome, SDHI implicitly and explicitly accepts certain elements of a Newtonian view of reality (i.e. reductionism, linearity and hierarchy) [2, 17]. An example of this reductionist approach is the descriptions of a single factor that influences health outcomes (e.g. socio-economic stratification of mortality due to asthma) and selecting interventions that focus on a single determinant (e.g. improving thermal comfort in houses that have inadequate warmth) [18]. Another assumption prevalent in this discourse is linearity, which assumes that determinants of inequalities can be applied across a wide range of contexts. For example, differential access to healthcare or education is explicitly or implicitly assumed to lead to variations in outcomes, almost in a linear fashion [6, 7, 17]. This view does not give adequate credit to unintended consequences commonly seen in reality. For example, mobile phones have improved connectivity, but their use while driving have become an important cause of road traffic accidents, a feature that was never predicted at the outset. Another key concept is the role of hierarchies or power, position and access to resources (e.g. in the understanding of socio-economic position). The concept of hierarchies is implicitly used to explain the process of SDHI as exemplified by terms such as proximate or distal determinants of health inequalities. This indicates a clear path of influences that arise ‘distal’ to the population group (e.g. labour laws that determine wage structure) and affect it through more ‘proximal’ factors that are closer to the population (e.g. income) [6, 7]. The statistical methods of estimating the effects of determinants also imply other features of the mechanistic reductionist paradigm. Earlier generation of studies used relatively simple statistical models such as Odds Ratios [19]. These methods assume that explanatory variables have a cause and-effect-pathway (i.e. unidirectional path of influences) and do not encompass positive or negative feed-back loops between the outcome variable and explanatory variables. For example, poor education leads to higher probability of ill-health, which combined together lowers the level of employment and ability to generate income, which in turn influences the ability to live in a more affluent neighbourhood and therefore reduces opportunities for further education, and higher grades of occupations, as well has increases exposure to more polluting environments. They also ignore interrelations among individuals. For example health education could have a positive health impact on an individual, that could indirectly improve the health of the individual’s friends. More sophisticated statistical methods, e.g. generalized linear models such as multiple regression, logistic regression and Poisson regression, account for multiple explanatory variables and to those that are not normally distributed. However, these analyses do not include feedback loops and interrelations among individuals. The latter require multilevel or hierarchical regressions models [20]. They ‘implicitly assume that these effects can be isolated from each other and do not allow for feedback loops or reciprocal interactions between groups and individuals, or between outcomes and predictors’ [21, 22].

## Visualizing SDHI from a systems science perspective

Systems science combines systems theory and complexity science. Systems theory states that properties of a complete system cannot be predicted by disaggregating, analyzing and exploring its individual constituent parts alone [23]. Complexity applies systems theory to open and adaptive systems (i.e. complex adaptive systems) and views health outcomes as an emergent property of such systems [1, 2]. The next sections propose a two-step process to visualize SDHI from a systems science perspective, firstly, by shifting away from a reductionist paradigm towards a systems approach and secondly, by enriching this with principles of complexity science.

In applying the systems approach, a point of departure from the conventional model of thinking is to consider population health outcomes as one of many components of the human condition or the ‘standard of living’ of a population. This human condition has several other facets or components such as educational attainment, economic well-being, and social status. These components too exhibit patterns of inequalities, in a manner similar to health outcomes. Health inequalities should be viewed as patterns in health outcomes that arise in association with other patterns of human condition, and lie within this milieu. Extricating health outcomes from these other human conditions and exploring it individually is therefore arbitrary, though justified on grounds of interest, existing disciplinarity and convenience of tackling one component at a time

SDHI can be visualized from a systems approach using a matrix that captures at least part of the elements of the human condition system. Each group of columns will represent a facet of the human condition (e.g. health outcomes, educational attainment). For comparison between countries, the parameters should be uniform and preferably continuous variables. The bundle of parameters in health outcomes could include life expectancy at birth (LEB), vaccination rate of children at 5 years, inverse of infant mortality rate (IMR), inverse of maternal mortality rates (MMR), and inverse of childhood malnutrition rates. Similarly, the parameter bundle on educational attainment could be percentage enrolment by 6 years, percentage in secondary level education, and per-capita investment in education, and for socio-economic status, the per-capita income, and employment rates. Each row could be for a particular year or for specific intervals (e.g. 2005 to 2010). An appropriate colour coding for the parameters will give rise to a pattern in the matrix that will be a fingerprint of the human condition of a particular country. The whole matrix represents the totality of the human condition as an output. A similar method of visualization is used to illustrate worldwide trends in life expectancy at birth from 1970 to 2010 published in the Human Development Report [24].

This process will also demonstrate the disadvantages of narrowing our perspective to a particular health outcome (e.g. obesity rates) or its determinants (e.g. physical activity). When such single parameters are selected from the matrix, we are in reality focusing on a single facet of health outcomes extracted from a column of the matrix. Instead, the preferred option is to express data on several other health related outputs such as IMR, MMR, LEB, and morbidity rates. This will immediately highlight unintended consequences, a feature of complex adaptive systems. Once a predetermined bundle of health outcomes are used to describe health, e.g. LEB, IMR, and MMR, comparisons between countries or regions become easier.

**Fig. 1:**
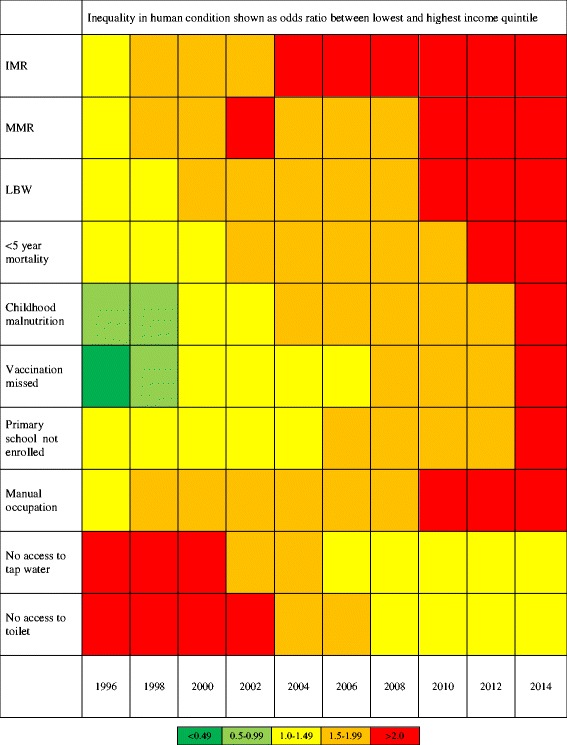
Inequalities in human condition from 1996 to 2014 as Odds Ratios

The second step is to incorporate an inequalities dimension to this form of visualization. This could be done on standardized measures of inequalities or stratifiers (e.g. across income or wealth or educational attainment, or geographic region) over a period or 10-20 years (see Fig. 1).

$$ \mathrm{Odds}\;\mathrm{Ratio}=\frac{\mathrm{Odds}\;\mathrm{of}\;\mathrm{parameter}\;\mathrm{in}\;\mathrm{poorest}\;\mathrm{in}\mathrm{come}\;\mathrm{quintile}}{\mathrm{Odds}\;\mathrm{of}\;\mathrm{parameter}\;\mathrm{in}\;\mathrm{richest}\;\mathrm{in}\mathrm{come}\;\mathrm{quintile}} $$

This will allow tracking of trends within a country. For example, a stratifier such as income could impact across several categories of the human condition. This impact could be visualized using the following summary measure of inequalities:

- Health inequalities: Odds Ratios between highest and lowest income quintile in parameters such as IMR, MMR, under-5 year vaccination rates, childhood nutrition, LEB
- Educational attainment: Odds Ratios between highest and lowest income quintiles in education such as primary school enrollment, secondary school enrollment, and tertiary education.
- Inequalities in social capital: Odds Ratios between highest and lowest income quintiles in measures of social capital, contact with social networks,

One option is for individual countries to have several of these charts, one for each stratifier, and track selected measures of inequalities (e.g. ratios of IMR, LEB, MMR, primary school enrollment) over a period (e.g. every 5 years). Each cell will be filled by the result of the Odds Ratio. In order to give an immediate visual impression, a simple set of rules could be observed to colour the cells in the matrix. The colour coding could be such that the cells showing extreme values of a ratio (e.g. above an Odds Ration of 2) and having the widest the inequalities will receive red colour and the least receive green, with other intermediates receiving light green, yellow, and orange. The precise values at which the colour transits would be arbitrary (e.g. one may choose a ratio of 2 and above to be red and below 0.5 to be green) See Fig. 1.

However, it is necessary to be consistent in order to allow for comparisons along the time axis and between countries or regions. A series of matrices developed for each stratifer in a country, could be analysed subsequently using hierarchical clustering algorithms (e.g. hierarchical clustering and k-means clustering) and computational methods (e.g. organizing maps and artificial neural networks) [25]. Hierarchical clustering will produce a ‘tree’ of those cells that are closest to each other (e.g. those with closest values as percentages). Heat maps can also be used to illustrate the clustering and it increasingly used to describe multimorbidity [26]. Such analyses from individual countries and the emerging patterns of health outcomes will give invaluable insight on the processes that are involved in giving rise to these particular patterns. It is possible that this method will demonstrate clusters of inequality measures that would not be expected from usual epidemiological parameters. An analogous situation was seen with data from acutely ill patients in Intensive Care Units, where by clusters of physiological parameters were observed that could not be defined by conventional knowledge [27].

The third step is to view the system as a dynamic entity and to include dynamic dimension to the approach. Describing outcomes at a given time is a mere snapshot of a dynamic process and analogous to prognostication of diabetes and its risks by using a single random blood glucose level. Extending this further, the predictions become more robust if a series of observations are made and this dynamism is incorporated into the model by using rates of change of parameters (e.g. rate of decline of IMR). Parallels are seen in systems medicine where serial analyses of the pulsatile secretion of insulin (rather than fasting insulin) was found to be an early feature of diabetes rather than a single value [28]. Data can now be gathered relatively inexpensively using crowd-sourcing techniques (i.e. gathering information by engaging with a large number of people usually via the Internet) and real-time surveillance of information in Twitter and other social media [29, 30].

### Implications of a systems science approach

A systems approach views the human condition and their determinants as causes as well as outcomes. They are interlinked in a causal web, rather than a linear path of social determinants leading to a range of health outcome. The feedback loops lead to outcomes influencing their causations. For example, low income and deprivation lead to poorer health outcomes, which in turn makes the group even poorer and worsening health outcomes. In order to operationalize the above, more sophisticated models such as systems dynamic models are required [31–33]. Analogous models are now being developed for specific disorders such as obesity [34].

A systems approach to SDHI emphasizes the role of the context in determining health inequalities. The relationships between the variations in health outcomes and other parameters would be more or less unique to that particular system. The interpretation of the outputs is heavily dependent on the context and less generalizable across different nations, social groups, or environments. This is yet another reason to explore natural experiments to identify causal pathways and interventions for a given context [21].

### Application of a complexity science approach to SDHI

The next advance is to incorporate complexity science to the systems perspective. This will require perceiving the patterns of human condition in a given population group to be an emergent property of a complex adaptive system (CAS). The latter is a special type of system that is ‘open’ to its environment, with non-linear interactions among a dynamic set of other interconnected systems and subsystems. Other systems include the political system (e.g. the predominant political governance system), the physical environment (e.g. pollution levels or transport), and biological systems (e.g. genetic predispositions), within the context of a larger social system [2]. One could position this within the context of global ecosystems (e.g. the biosphere, the atmosphere, the hydrosphere, geosphere and cryosphere).

A feature of a CAS is that it has emergent properties, i.e. it is almost impossible to predict precisely what it the larger system will evolve to, by using knowledge relating to the interactions among its components and sub-components [35]. Rather than mathematical modeling where equations are the basis of the model, some complexity scientists use simulations using computer-based programmes (e.g. Python) [36]. The building blocks of these simulation models are considered to be as fundamental and irreducible as mathematic equations, and are being hailed as a ‘new kind of science’ [37].

If the analogy of the matrix of SDHI used in the previous section is applied, the picture will be one of continuously changing colours and patterns that are not predictable. Complexity scientists would mimic these patterns through computer simulations using simple rules that describe the inter-relationships among parameters. Of the commonly used computer simulations, those most likely to mimic patterns of health outcomes seen in the matrix model are agent-based models, cellular automata, and networks.

Initial work on agent-based models by Schelling showed that segregation in a city (often attributed to racial discrimination) can be simulated by simple rules applied to a grid where each cell represents a house [38]. Consider an example where 90 % percent of houses are occupied randomly, either by blue or red, in approximately equal proportions. A programme will simulate future scenarios if they follow two simple rules: place each house where at least 2 of 8 encircling them are of similar colour as the house, and avoid the cell if they have one or none of same colour. Within a few iterations, the emerging model begins to mimic cities that are segregated by race, poverty, or social status. These simulations provide evidence (but no proof) that several alternate pathways could potentially explain urban clusters of race or poverty, rather than discrimination. Extrapolating to health outcomes, they challenge area-wise aggregation of health outcomes are through pathway such as the neighbourhood effects or the effects of urbanization on health outcomes [38].

Cellular automata is another simulation where the programme places lines of square cells next to each other (e.g. you fill the cell with a black cell, if two of the eight cells around the cell of interest are black) as in a checker board [37, 39]. The interesting observations include the following: minor changes in the rules and initial conditions lead to emergence of widely divergent patterns, some lead to recurring patterns, and similar patterns arise with different starting points and different set of rules. If this metaphor is applied to SDHI, it illustrates the uniqueness of the context to a particular pattern of health outcomes in a population, and the theoretical possibility that widely divergent determinants lead to similar patterns of outcomes in different situations.

Networks perceive systems as interlinked webs. The strengths and numbers of links among nodes can be varied to generate emergent properties mimicking reality. Milgram pioneered the concept of small world networks, in which the degrees of separation between two individual members is considerably small compared to the given population [40]. This was illustrated by an experiment that showed an average six intermediate acquaintances separate any two people on the planet. Thus a highly infectious disease could theoretically spread to all 7 billion on the globe in about six incubation periods! Another improvement of this simulation is to incorporate the highly connected super-spreaders who are disproportionately relevant to rapid spread of the disease, called scale-free networks [41]. In this model, new members of the network connect preferentially to those who already have a large number of contacts (i.e. analogous to individuals befriending the most popular members of a network). The resulting number of contacts per individual takes a power-law distribution. The recent epidemic of Severe Acute Respiratory Syndrome (SARS) was such an example where a significant proportion of the infections were due to ‘super-spreaders’ [42]. These networks resemble patterns observed in the spread of information along social networks and obesity in geographic areas giving rise to amazing patterns of spread [43].

## Conclusions

Characterizing outcomes of the human condition (including health inequalities) as patterns that emerge in a complex adaptive system has several implications. Firstly, isolating health outcomes from the other human conditions are problematic because they are features or dimensions of a larger picture and therefore more sophisticated forms of systems analyses will be required to shed light on SDHI. Secondly, emerging patterns of a complex adaptive system cannot be estimated with precision by using the known interactions among its components (such as economic development, political freedom, health system, culture etc.). Different combinations of the same bundle of factors or determinants can give rise to similar patterns or outcomes (i.e. property of convergence), and minor variations in the initial condition could give rise to widely divergent outcomes. This questions the ability to generalize with precision, the determinants of health outcomes from one contextual environment to another. Thus the favoured models would be those developed for the particular context using longitudinal data and using systems dynamics of feedback and interrelationships. Finally, computer simulation models (e.g. agent-based models) would shed light on possible mechanisms as to how factors or determinants interact and lead to emergent patterns of health outcomes of populations. The metaphor needs to shift from the current visualizations of health outcomes as rigid bar diagrams demonstrating the step-wise gradient to a more vibrant multi-coloured canvass with changing hues.

## Abbreviations

SDH: Social Determinants of Health
SDHI: Social Determinants of Health Inequalities
CAS: Complex Adaptive System
CSDH: Commission on Social Determinants of Health
IMR: Infant Mortality Rates
LEB: Life Expectancy at Birth
MMR: Maternal Mortality Rates
SARS: Severe Acute Respiratory Syndrome
